# Antimicrobial Resistance and Risk Factors of Canine Bacterial Skin Infections

**DOI:** 10.3390/pathogens14040309

**Published:** 2025-03-24

**Authors:** Qian Wang, Siyu Chen, Shizhen Ma, Ying Jiao, Huiyi Hong, Siying Wang, Wei Huang, Qi An, Yu Song, Xukun Dang, Gege Zhang, Haiqin Ding, Yang Wang, Zhaofei Xia, Lu Wang, Yanli Lyu

**Affiliations:** 1 College of Veterinary Medicine, China Agricultural University, Beijing 100193, China; bs20223050493@cau.edu.cn (Q.W.); siyuchen@cau.edu.cn (S.C.); bs20223050484@cau.edu.cn (Q.A.); sy1296328095@126.com (Y.S.); 13051557166@163.com (G.Z.); 19807487540@163.com (H.D.); drxia@126.com (Z.X.); 2 Beijing Zhongnongda Veterinary Hospital Co., Ltd., Beijing 100193, China; jiaoying0617@126.com (Y.J.); hunghuiyi@hotmail.com (H.H.); wangsy0161@163.com (S.W.); kennawei@126.com (W.H.); 3 National Key Laboratory of Veterinary Public Health and Safety, College of Veterinary Medicine, China Agricultural University, Beijing 100193, China; shizhenma@cau.edu.cn (S.M.); d99@cau.edu.cn (X.D.); wangyang@cau.edu.cn (Y.W.); 4 Key Laboratory of Animal Antimicrobial Resistance Surveillance, Ministry of Agriculture and Rural Afairs, College of Veterinary Medicine, China Agricultural University, Beijing 100193, China

**Keywords:** canine bacterial skin infections, antimicrobial resistance, risk factors, *Staphylococcus pseudintermedius*, methicillin resistance

## Abstract

Bacterial skin infections are common in dogs and often secondary to underlying conditions like allergies or ectoparasite infestations. Untreated primary causes can lead to recurrent infections and an increased risk of antimicrobial resistance, including methicillin-resistant *Staphylococcus pseudintermedius* (MRSP), posing a substantial clinical challenge. Here, we analyzed 896 canine bacterial skin infection samples collected from the China Agricultural University Veterinary Teaching Hospital between 2018 and 2022. Species identification was confirmed by MALDI-TOF and 16S rRNA gene sequencing. Of the 896 samples, 722 (80.6%) yielded 1123 bacterial isolates, with *Staphylococcus pseudintermedius* (*n* = 421), *Pseudomonas aeruginosa* (*n* = 108), and *Escherichia coli* (*n* = 73) being the most prevalent. Antimicrobial susceptibility was evaluated using the broth microdilution method according to CLSI guidelines. Notably, resistance to florfenicol in *S. pseudintermedius* increased from 9.1% in 2018 to 20.0% in 2022, while resistance to ceftriaxone in *E. coli* rose from 30.0% to 72.7% over the same period. Among 305 reviewed cases, pyoderma (47.5%, 145/305) was the most common infection type, predominantly associated with *S. pseudintermedius* (*n* = 114), followed by otitis (25.6%, 78/305) primarily linked to *P. aeruginosa* (*n* = 24). Mixed infections occurred in 35.4% (108/305) of cases, with *S. pseudintermedius* as the most frequently isolated species in both single and mixed infections. The multivariable logistic regression model revealed that MRSP infections were correlated with a history of invasion (*p* <0.001) and prolonged disease duration (six months to less than one year: *p* = 0.005; one year or longer: *p* < 0.001). Core-genome SNP analysis showed that eight dogs were infected with identical *S. pseudintermedius* strains, in which one dog exhibited a shift from gentamicin susceptibility to resistance within nine days. Conversely, three dogs were infected by distinct *S. pseudintermedius* strains at two time points. To effectively manage MRSP infections and chronic skin infections in dogs, rigorous disinfection protocols in veterinary hospitals, control of disease duration, prevention of recurrent infections, and continuous monitoring of antibiotic resistance patterns are essential.

## 1. Introduction

The skin is the largest organ in both animals and humans, serving as a complex barrier composed of microbial, chemical, physical, and immune defenses. Disruptions to this barrier can lead to various dermatological conditions, including infections, inflammation, allergies, and even cancer [1]. Due to its direct exposure to the environment, the skin is highly susceptible to bacterial invasion. Among bacterial skin infections in dogs, pyoderma is the most prevalent and often occurs secondary to underlying conditions such as allergies or ectoparasite infestations [2]. These infections are a primary reason for antimicrobial prescriptions in veterinary medicine [3]. While not typically life-threatening, failure to address the underlying cause can lead to recurrent infections, exacerbating the risk of antimicrobial resistance (AMR) [4].

Given the high prevalence and recurrence of bacterial skin infections in dogs, understanding the characteristics of these pathogens and their resistance profiles is essential. *Staphylococcus pseudintermedius*, an opportunistic pathogen commonly found in healthy dogs, is the most frequently identified bacterium in canine bacterial skin infections [5]. Other skin infection-associated pathogens, such as *Pseudomonas aeruginosa* and *Escherichia coli*, are often linked to more complicated infections [2,6]. The emergence of multidrug-resistant (MDR) pathogens has further complicated antimicrobial therapy in veterinary medicine [7]. Companion animals, including dogs and cats, are recognized as potential reservoirs of AMR bacteria [8]. Although *S. pseudintermedius* rarely causes infections in humans, transmission, particularly in individuals in close contact with companion animals, has been documented [9], leading to its classification as a zoonotic pathogen [10,11].

Methicillin resistance in *S. pseudintermedius*, similar to methicillin-resistant *Staphylococcus aureus* (MRSA), is mediated by the *mecA* gene, which is located on a mobile genetic element known as the Staphylococcal chromosomal cassettes mec (*SCCmec*). This genetic element facilitates horizontal gene transfer among different *Staphylococcus* species, conferring resistance to β-lactam antibiotics [12,13,14]. Previous research has indicated that dogs with surgical site infections or skin and soft tissue infections are at a higher risk of MRSP infection than those with infections at other body sites [15]. This underscores the importance of antimicrobial susceptibility testing in guiding appropriate treatment for canine bacterial skin infections. Despite their clinical significance, studies investigating bacterial isolates from canine skin infections remain limited, particularly in tracking AMR trends. Surveillance of antimicrobial resistance patterns is essential for informing treatment strategies and mitigating the spread of resistance in veterinary dermatology. Therefore, this study aims to (1) investigate the epidemiology of bacterial pathogens associated with canine bacterial skin infections, (2) assess their AMR profiles, (3) identify risk factors for MRSP infection, and (4) explore the adaptive evolution of *S. pseudintermedius* in canine hosts.

## 2. Materials and Methods

### 2.1. Sample and Isolates Collection

Canine bacterial skin infection samples were obtained from the clinical laboratory of the China Agricultural University Veterinary Teaching Hospital between January 2018 and December 2022. Bacterial skin infection was diagnosed based on clinical and cytological findings. The CARPet system was employed to collect and recover bacterial isolates, and their antimicrobial susceptibility was tested and analyzed [16]. All isolates were cultured on Brain Heart Infusion agar containing 5% defibrinated sheep blood and incubated at 37 °C for 24 h. Species identification was confirmed using matrix-assisted laser desorption ionization–time of flight mass spectrometry (MALDI-TOF MS, Autobio, Zhengzhou, China) and/or 16S rRNA gene sequencing.

### 2.2. Antimicrobial Susceptibility Testing

Antimicrobial susceptibility testing was performed on the three most frequently isolated bacterial pathogens from the canine bacterial skin infection samples: *S. pseudintermedius*, *P. aeruginosa*, and *E. coli*. The broth microdilution method was used with custom-made microdilution panels (Thermo Fisher Scientific, Waltham, MA, USA), following the guidelines in the Clinical and Laboratory Standard Institute (CLSI) M07 document [17]. For *S. pseudintermedius*, 12 antimicrobial agents were tested, including oxacillin, doxycycline, gentamicin, enrofloxacin, azithromycin, florfenicol, fusidic acid, trimethoprim-sulfamethoxazole, rifampin, linezolid, vancomycin, and daptomycin. For *P. aeruginosa*, seven were tested, including amikacin, gentamicin, cefquinome, colistin, levofloxacin, enrofloxacin, and meropenem. For *E. coli*, 12 agents were tested, including ceftriaxone, cefquinome, meropenem, gentamicin, amikacin, levofloxacin, enrofloxacin, trimethoprim-sulfamethoxazole, tigecycline, florfenicol, colistin, and doxycycline. *S. aureus* ATCC 29213, *P. aeruginosa* ATCC 27853, and *E. coli* ATCC 25922 served as the quality control strains. Results were interpreted according to the clinical breakpoints defined the CLSI VET01S [18] and CLSI M100 [19] guidelines. Data analysis was performed using the WHONET software (version 2022). Cochran–Armitage trend test was applied to evaluate the resistance trends from 2018 to 2022, with a *p*-value of less than 0.05 considered statistically significant.

### 2.3. Retrospective Analysis of Risk Factors for MRSP Infection

We collected clinical information for each dog, including age, sex, and sampling date, as well as medical treatment details such as disease duration, history of surgery, history of invasion (defined as any event or procedure that compromises the skin barrier, such as bite wounds, punctures, or other traumatic injuries), and history of hospitalization. Additionally, data on the number of bacterial cultures and species identification were also collected. Only cases with complete clinical data were included in the retrospective analysis to identify risk factors for MRSP infection. Univariate statistical analysis was performed using the Chi-square test in SPSS 26.0. For multivariate analysis, all variables were included in a logistic regression model, followed by stepwise selection using the Akaike Information Criterion (AIC) to identify the best model, with 95% confidence intervals (CIs) calculated for each variable.

### 2.4. Whole Genome Sequencing of S. pseudintermedius and Bioinformatics Analysis

Given that *S. pseudintermedius* is the primary pathogen in canine bacterial skin infections, we aimed to determine whether it undergoes adaptive evolution in response to chronic infection pressures. We collected *S. pseudintermedius* isolates from 10 dogs (designated SP1 to SP10), all of which underwent repeated sampling, with *S. pseudintermedius* isolated from each dog. Genomic DNA from *S. pseudintermedius* isolates was extracted using the HiPure Bacterial DNA Kit (Magen, Guangzhou, China). Libraries were prepared with the TruSeq Nano DNA High Throughput Library Prep Kit (Illumina, San Diego, CA, USA) and sequenced on the Illumina NovaSeq Xplus platform. Raw sequence data were assembled using SPAdes (v.3.14.0) [20] via the Unicycler (v.0.5.0) assembly pipeline [21]. The assembled genomes were deposited in NCBI (BioProject accession number PRJNA1229125). A total of 21 genomes were used to generate a core-genome single nucleotide polymorphism (SNP) alignment and to construct a phylogenetic tree using Parsnp (v.2.0.2) in the Harvest package [22]. The phylogenetic tree was midpoint-rooted and annotated in ITOL (https://itol.embl.de/, accessed on 27 January 2024). Sequence types (STs) were identified using SRST2 (v.0.2.0) [23]. The genomes were further analyzed using Abricate (v.1.0.1) (https://github.com/tseemann/abricate, accessed on 16 January 2024) against the NCBI database [24] and the Virulence Factor Database [25] to identify antibiotic genes (ARGs) and virulence factors (VFs). Mutations were identified using the Breseq computational pipeline (v.0.39.0) [26], and comparative genomic visualization was performed using Easyfig (v2.2.5) [27].

### 2.5. RNA Extraction and RT-qPCR Analysis of aacA-aphD Expression

Total RNA was extracted from *S. pseudintermedius* isolates from dog SP7 using the Hibind bacterial RNA kit (ZENPIO, Shanghai, China), following the manufacturer’s protocol. One microgram of purified RNA was reverse transcribed into cDNA using the RT Master Kit (Takara, Kusatsu, Japan). The PCR reaction mixture was prepared using the Taq Pro Universal SYBR qPCR Master Mix (Vazyme, Nanjing, China) according to the manufacturer’s instructions. Real-time qPCR was performed in triplicate using the QuantStudio™ 7 Flex system (Applied Biosystems, Foster City, CA, USA). The amplification conditions were set as per the manufacturer’s instructions for the SYBR qPCR Master Mix. The expression levels of *aacA-aphD* were normalized to the 16s rRNA gene as the reference, and changes in gene expression were calculated using the comparative CT method. Primers used for the PCR are listed in Table S1 and were synthesized by Sangon Biotech (Shanghai, China).

## 3. Results

### 3.1. Isolation of Bacterial Strains and Antimicrobial Resistance Analysis

**Figure 1:**
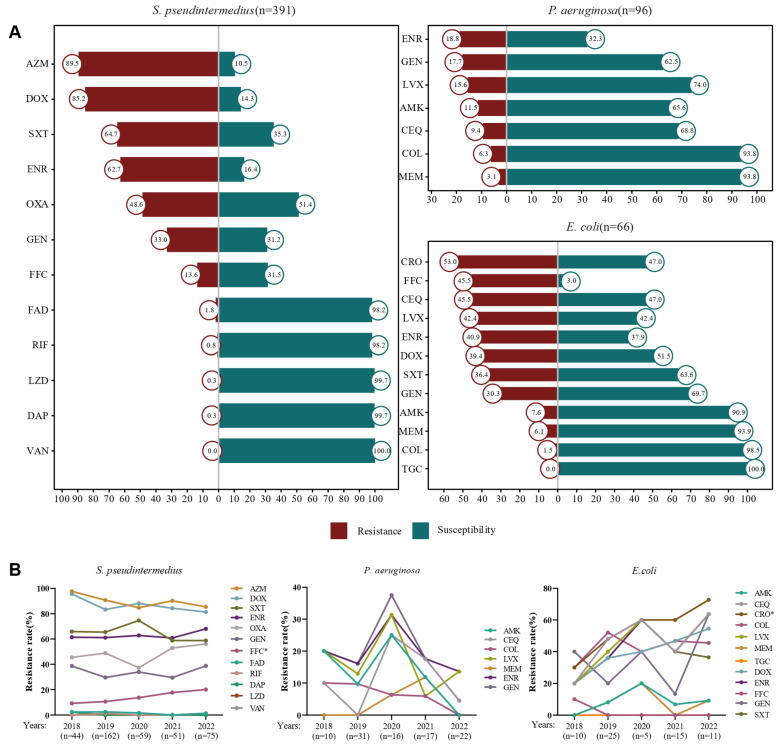
AMR rates of the top three isolates from canine bacterial skin infection samples. (**A**) Diverging bar plots showing the AMR rates and (**B**) Line charts showing the variation of resistance rates in tested isolates from 2018 to 2022. Oxacillin (OXA), azithromycin (AZM), daptomycin (DAP), doxycycline (DOX), enrofloxacin (ENR), fusidic acid (FAD), florfenicol (FFC), gentamicin (GEN), linezolid (LZD), rifampin (RIF), trimethoprim-sulfamethoxazole (SXT), vancomycin (VAN), amikacin (AMK), cefquinome (CEQ), colistin (COL), levofloxacin (LVX), meropenem (MEM), gentamicin (GEN), ceftriaxone (CRO), and tigecycline (TGC). *p* < 0.05 (*) is considered as statistically significant.

A total of 896 canine skin infection samples were submitted to the medical microbiology laboratories at the China Agricultural University Veterinary Teaching Hospital between 2018 and 2022. Of these, 19.4% (*n* = 174) were negative for microbial growth. From the remaining 722 samples, 1123 isolates were recovered, representing 126 bacterial species (Table S2). The most frequently identified pathogens were *S. pseudintermedius* (37.5%, 421/1123), *P. aeruginosa* (9.6%, 108/1123), and *E. coli* (6.5%, 73/1123) (Table S3). However, due to insufficient growth during isolate recovery, the antimicrobial susceptibility testing was conducted on *S. pseudintermedius* (*n* = 391), *P. aeruginosa* (*n* = 96), and *E. coli* (*n* = 66). Resistance rates for *S. pseudintermedius* exceeded 80% for azithromycin and doxycycline, while resistance to oxacillin, enrofloxacin, and trimethoprim-sulfamethoxazole ranged from 48.6% to 64.7% (Figure 1A). Notably, florfenicol resistance in *S. pseudintermedius* showed a significant increase from 9.1% in 2018 to 20.0% in 2022 (*p* < 0.05) (Figure 1B). The resistance rates of *P. aeruginosa* to the tested antimicrobial agents were generally below 20.0%, with the highest resistance observed for enrofloxacin (18.8%) (Figure 1A). Interestingly, resistance rates for *P. aeruginosa* peaked in 2020 but decreased in the subsequent years (Figure 1B). For *E. coli*, resistance rates to florfenicol, cefquinome, and ceftriaxone ranged from 45.5% to 53.0% (Figure 1A), and resistance to ceftriaxone showed a significant increase from 30.0% in 2018 to 72.7% in 2022 (*p* < 0.05) (Figure 1B).

### 3.2. Retrospective Analysis of Canine Bacterial Skin Infection Cases

**Figure 2:**
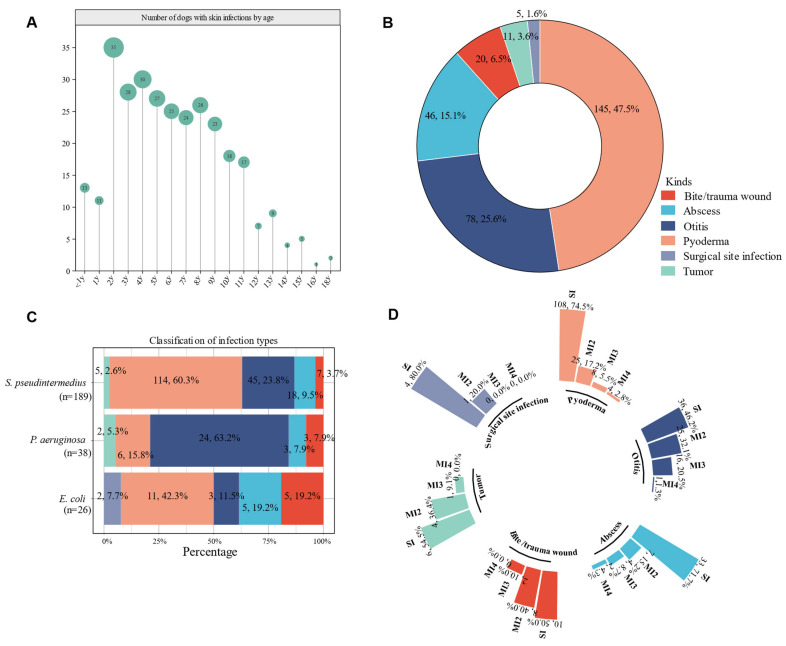
Details of canine bacterial skin infection cases. (**A**) Age distribution. (**B**) Proportion of different infection types. (**C**) Proportion of each infection type caused by the top three isolates. (**D**) The occurrence of mixed infections in each infection type. Single bacterial infection (SI), two-species infections (MI2), three-species infections (MI3), and four-species infections (MI4).

A retrospective analysis was performed on 305 cases with complete clinical information. The data revealed that male dogs were significantly more affected than females (66.6% vs. 33.4%), with dogs aged 2 years being the most commonly affected (median age: 5 years, Figure 2A). Pyoderma was the most common infection type (47.5%, 145/305), followed by otitis (25.6%, 78/305), abscess (15.1%, 46/305), and bite/trauma wounds (6.6%, 20/305, Figure 2B). The most frequently isolated bacterium was *S. pseudintermedius* (41.3%, 189/458), followed by *P. aeruginosa* (8.3%, 38/458) and *E. coli* (5.7%, 26/458) (Table S4). Analysis of infection types showed that *S. pseudintermedius* was most frequently isolated from pyoderma (60.3%, 114/189), whereas *P. aeruginosa* was primarily associated with otitis (63.2%, 24/38) (Figure 2C).

Regarding bacterial infection types, single bacterial infections accounted for 64.6% (197/305) of cases, with 64.5% (127/197) of these attributed to *S. pseudintermedius*. Mixed bacterial infections were observed in 35.4% (108/305) of cases, with two-species infections in 23.0% (70/305), three-species infections in 10.2% (31/305), and four-species infections in 2.3% (7/305) (Table S5). Notably, *S. pseudintermedius* was the most frequently isolated bacterium in mixed infections involving two or more species. Further analysis revealed that surgical site infections, pyoderma, and abscesses had higher rates of single-bacterial infections (71.7–80%), while otitis, bite/trauma wounds, and tumors had higher rates of mixed infections, reaching around 50% (Figure 2D).

### 3.3. Risk Factors for MRSP Infections Were History of Invasion and Disease Duration

**Figure 3:**
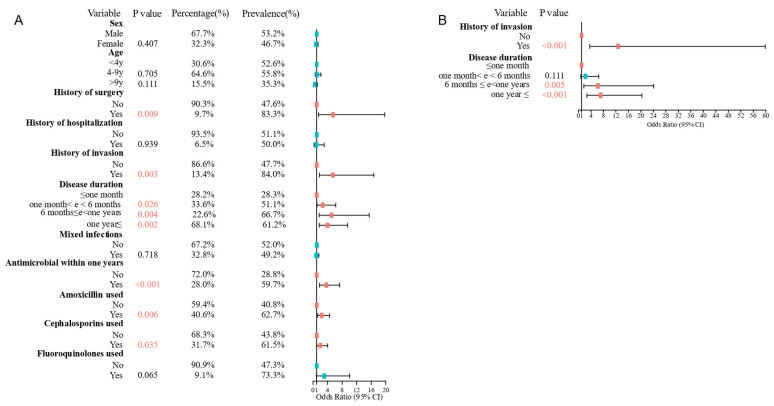
Forest plots of (**A**) univariate and (**B**) multivariate analyses of the risk factors associated with MRSP infection in dogs with skin bacterial infection. Red boxes indicate statistically significant differences, while blue boxes indicate no statistically significant differences.

The risk factors for MRSP infections in canine skin were assessed by documenting basic patient information, medical treatment details, and infection history, including disease duration, history of surgery, history of invasion, and history of hospitalization. Complete case information was available for 186 *S. pseudintermedius* isolates, of which 95 were identified as MRSP isolates. Statistical analysis revealed that history of surgery, history of invasion, disease duration, antimicrobial usage within one year, usage of amoxicillin, and usage of cephalosporin were significantly associated with MRSP infections (Figure 3A). A logistic regression model was constructed with all variables from the univariate analysis, stepwise AIC selection was used to determine the best model, which included two variables: history of invasion and disease duration. Dogs with a history of invasion (OR = 12.74 95% CI: 3.747–59.8, *p* < 0.001) or longer disease duration (6 months to less than one year: OR = 6.284, 95% CI: 1.812–24.003, *p* = 0.005; one year or longer: OR = 7.111, 95% CI: 2.703–20.313, *p* < 0.001) were more likely to be infected with MRSP (Figure 3B).

### 3.4. Genomic and Phenotypic Insights into Antimicrobial Resistance in S. pseudintermedius

**Figure 4:**
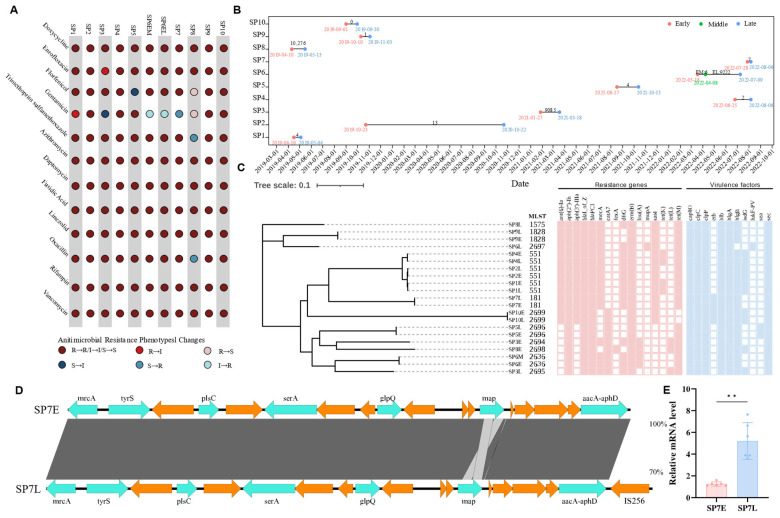
Overview of *S. pseudintermedius* isolation and resistance analysis from canine samples. (**A**) Phenotypic antimicrobial resistance changes in isolates from individual dogs over time. (**B**) Sampling timeline for each dog. Early (first sampling), Middle (second sampling; only SP6), and Late (second sampling; third sampling in SP6). (**C**) Core-genome phylogenetic tree of 21 *S. pseudintermedius* isolates, with resistance genes and virulence factors. (**D**) Comparative genomic alignment of the contig containing IS256, generated using Easyfig, blue arrows indicate gene, and orange arrows represent coding sequences with functional annotations that include both well-characterized proteins and hypothetical proteins. (**E**) RT-qPCR analysis of *aacA-aphD* expression. Isolates ending with “E” represent the first isolates of *S. pseudintermedius*, while those ending with “L” correspond to second isolates (in dog SP6, isolates ending with “M” indicate the second isolates and those ending with “L” refer to the third isolates). *p* < 0.01 (**) is considered as statistically significant.

Repeated samplings were conducted on 10 dogs (SP1–SP10), with *S. pseudintermedius* isolated from all of them, including one dog (SP6) that underwent three samplings (Figure 4B). Phenotypic analysis of AMR revealed that most isolates from different time points showed no significant changes, although a few exhibited variations (Figure 4A). Whole-genome sequencing was performed on 21 *S. pseudintermedius* isolates, and a core-genome phylogenetic tree was constructed. Core-genome SNP analysis revealed that six isolates from SP8, SP3, and SP6 (two isolates from the second and third samplings) showed substantial SNP differences, suggesting infection by distinct strains (Figure 4C). In contrast, 16 isolates from eight dogs exhibited SNP counts ranging from 0 to 13 (including two isolates from the first and second samplings of SP6), indicating that these dogs were infected by the same strain. Notably, two isolates from dog SP7 displayed a shift from gentamicin susceptibility (MIC = 4 mg/L) to resistance (MIC = 16 mg/L) within 9 days between the two samplings. However, no significant changes in ARGs or VFs were detected between the two isolates, and the core-genome SNP count was limited to 7. Further analysis revealed that the second isolate contained an IS*256* element inserted downstream of the *aacA-aphD*, mutations in the *rpoC* (T204P), *dnaE* (P47L), and *menD* (V156A) genes, as well as a number of changes in the genes encoding various hypothetical proteins (Figure 4D, Table S6). qPCR results showed a significant increase in *aacA-aphD* expression in the second isolate from dog SP7 (Figure 4E), suggesting that the emerging gentamicin resistance may be associated with the enhanced expression of *aacA-aphD*.

## 4. Discussion

Bacterial skin infections are prevalent in dogs and are one of the primary reasons veterinarians prescribe antimicrobial agents for treatment [3]. A study conducted in Bulgaria (2019–2023) revealed that the highest percentage of MDR was detected among isolates from suppurating wounds and abscesses [28]. Data from our CARPet system showed that skin-derived bacterial isolates rank second among all sampling sites. These findings highlight the critical need for AMR surveillance of isolates from canine skin infections. Our study represents the most comprehensive surveillance of bacterial isolates and AMR patterns in canine bacterial skin infections in China to date; *S. pseudintermedius*, *P. aeruginosa*, and *E. coli* identified as the most common bacterial species from canine skin infections are consistent with findings from similar studies in Italy (2016–2019) [29]. In addition, the 2013–2014 European ComPath project surveillance also identified *Streptococcus* spp. as the major bacterial species responsible for skin infections [30]. The direct exposure of skin to external microbes frequently leads to coinfections. The rate of coinfection (35.4%) in this study was higher than that reported in Italy (16%) [29]. However, other studies have observed much higher coinfection rates, ranging from 61.7% [31] to 80% [32] in cases of otitis in dogs. The observed variations in coinfection rates may reflect differences in sampling protocols, geographical locations, and study methodologies. Regardless, the high prevalence of coinfections in canine skin and otitis infections emphasizes a need for deeper study into the interactions between pathogens and their role in pathogenesis.

*S. pseudintermedius* was the most frequently isolated bacterium in this study, underscoring its critical role in canine bacterial skin infections. Over 80% of *S. pseudintermedius* isolates in this study showed resistance to azithromycin and doxycycline, raising concerns particularly for doxycycline, which is commonly used as a second-tier treatment for canine superficial pyoderma [33]. In addition, MRSP, which mediated a resistance to all β-lactam antibiotics, presents a considerable zoonotic risk, making it a critical challenge for veterinary clinical practice [34,35,36]. The high MRSP rate (48.6%) observed in this study is consistent with similar studies conducted in Korea (41.2%) [34] and Italy (41.0%) [29], but significantly higher than resistance rates reported in Portugal (31.0%) [37]. Our findings revealed that dogs with a history of invasion were more likely to develop MRSP infections. This is likely due to the ability of *S. pseudintermedius* to persist in the environment and its strong adhesion to canine keratinocytes [38]. Invasive procedures may facilitate the transmission of MRSP to animals during hospital visits. Previous studies have shown the potential transmission of MRSP within animal hospitals, involving diseased dogs, their owners, and veterinary staff [39]. These results emphasize the importance of implementing proper disinfection protocols in veterinary hospitals to prevent MRSP transmission. Furthermore, dogs with a longer disease duration (i.e., six months to a year or more) had an increased risk of MRSP infection. This association may be due to the frequent hospital visits and antibiotic treatments required for chronic conditions, which increases the likelihood of MRSP transmission [40]. This finding emphasizes the importance of controlling disease duration and preventing recurrent infections, especially in dogs with underlying conditions that predispose them to chronic infections.

Another important finding was the increased resistance to florfenicol in *S. pseudintermedius*, from 9.1% in 2018 to 20.0% in 2022. Similar increases have been reported in *E. coli* isolates from pigs and chickens in China (2008–2015) [41]. Although florfenicol is approved for topical treatment of otitis in pets, it is mainly used in food animals, with its usage increasing from 2018 to 2021 [42]. The observed increase in florfenicol resistance in pet isolates may be explained by the spread of resistance from food animals to pets, possibly through raw pet diets and commercial pet foods [43,44]. For instance, florfenicol-resistant *Enterococcus faecium* isolates from dogs and food sources (beef, eggs, and chicken) exhibit close genetic relationships [45].

Due to its intrinsic resistance to many antibiotics [46], *P. aeruginosa* is a challenging opportunistic pathogen to treat, frequently identified in cases of otitis among the 305 canine skin infection cases we reviewed. Additionally, our results showed that the resistance rates of *P. aeruginosa* to all tested antibiotics remained below 20%. This contrasts with higher resistance rates reported in other studies involving *P. aeruginosa* from dogs with skin infections or otitis, such as enrofloxacin (26.0%) in Brazil [47], gentamicin (62.06%), amikacin (55.17%), and meropenem (74.13%) in Romania [48]. This suggests that there are significant regional variations in AMR patterns, highlighting the importance of enhancing global surveillance of AMR to effectively control the spread and development of resistance in *P. aeruginosa*. For *E. coli*, the increase in ceftriaxone resistance (from 30% in 2018 to 72.7% in 2022) observed in our study contrasts with the significant decline in cefotaxime resistance in *E. coli* from pigs between 2011 and 2021 (from 21.4% to 4.1%) [42]. This discrepancy may be attributed to ceftriaxone being a commonly used antimicrobial agent for systemic infections in dogs [49]. Additionally, the Chinese Pet Anti-Infective Drugs Market Survey Report indicates that the annual sales volume of pet antimicrobial agents in China grew from 4,919,000 units in 2016 to 10,505,000 units in 2021 [50]. In contrast, following the China National Action Plan for Combating Animal Antimicrobial Resistance in 2017, antimicrobial use in China of food animals decreased by 9190.7 tons from 2017 to 2020 [51]. However, due to the limited number of *P. aeruginosa* and *E. coli* isolates, additional data from a larger sample size are needed to validate these trends and better understand the dynamics of AMR in these pathogens.

Since *S. aureus* has shown adaptive evolution in patients with atopic dermatitis [52], we hypothesized that similar mechanisms may apply to *S. pseudintermedius* in dogs with chronic infections. For the first time, we analyzed multiple *S. pseudintermedius* isolates from the same dogs and conducted a comparative genomic analysis. The majority of dogs were persistently infected by the same strain, with only three dogs exhibiting infections with distinct *S. pseudintermedius* strains. Notably, dogs with atopic dermatitis carried identical or closely related isolates at multiple sampling sites, while healthy dogs rarely did so, as reported previously [53]. These results suggest that chronic infections in dogs may favor a particular bacterium to occupy an ecological space within the host. However, the strain replacement in three dogs in our study highlights the importance of considering strain dynamics in clinical management, as shifts in bacterial populations may largely impact treatment efficacy and disease outcomes. For instance, one dog showed a shift from gentamicin susceptibility to resistance within 9 days between the two samplings despite no changes in the ARGs or VFs of two *S. pseudintermedius* isolates. However, the second isolate exhibited IS*256* insertion, *rpoC*, *dnaE*, and *menD* gene mutations, and a significant increase in *aacA-aphD* gene expression. The IS*256* is commonly found in multidrug-resistant enterococci and staphylococci [54], and the associations between IS*256* and resistance to gentamicin, amikacin, oxacillin, and ceftiofur have been reported in *S. pseudintermedius* [55]. Additionally, *rpoC* mutations have been linked to resistance to rifampin and oxacillin in *S. aureus* [56,57]. The mutations of the *dnaE* and *menD* gene may impact the DNA replication process [58] and the bacterium’s growth ability [59], but there have been no reports directly linking mutations in these genes to antibiotic resistance in *Staphylococci*. Thus, the observed alteration in gentamicin resistance is likely linked to the IS*256* insertion or *rpoC* mutation, which may lead to an increase in the *aacA-aphD* expression. However, further studies are needed to elucidate the precise role of these genetic changes in antibiotic resistance phenotypes and their clinical implications. Furthermore, we detected key VFs in all isolates, including *cap8O*, *clpC*, *clpP*, *hlb*, *hlgA*, and *sec*. This suggests that these VFs may play a critical role in the pathogenesis of *S. pseudintermedius* in canine skin infections. For example, previous studies have indicated that the *sec* enterotoxin isolated from *S. pseudintermedius* in dogs with pyoderma exhibits unique properties compared to other staphylococcal enterotoxins, including the ability to induce vomiting and T-cell proliferation [60]. Moreover, consistent with our findings, Izabel’s [61] study reported that all *S. pseudintermedius* isolates from dogs with pyoderma, otitis externa, and urinary tract infections also harbored *hlb*, while *clpC* and *clpP* have been implicated in cell formation and maintenance in *S. aureus* [62]. These findings underscore the potential importance of these virulence factors in sustaining chronic infections. However, further studies are needed to elucidate the specific mechanisms by which these VFs contribute to the development and persistence of skin infections.

In summary, we present the current AMR trends in canine bacterial skin infections in China, highlighting the increasing resistance of *S. pseudintermedius* to florfenicol and *E. coli* to ceftriaxone. To effectively combat MRSP infections in pets, it is imperative to implement comprehensive cleaning and disinfection protocols in animal hospitals, and control disease duration, particularly in dogs with underlying conditions. In addition, monitoring strain dynamics and detecting changes in antibiotic resistance are essential for managing long-term, chronic bacterial skin infections.

## Data Availability

The genome assemblies of *Staphylococcus pseudintermedius* have been deposited in the National Center for Biotechnology Information (NCBI) under BioProject accession no. PRJNA1229125. The study protocol and detailed data analysis plans are available from the corresponding author upon reasonable request. Furthermore, specific *Staphylococcus pseudintermedius* isolates used in this study are available upon request.

## Supplementary Materials

The following supporting information can be downloaded at: https://www.mdpi.com/article/10.3390/pathogens14040309/s1.
